# *Phyllolobium chinense* Fisch Flavonoids (PCFF) Suppresses the M1 Polarization of LPS-Stimulated RAW264.7 Macrophages by Inhibiting NF-κB/iNOS Signaling Pathway

**DOI:** 10.3389/fphar.2020.00864

**Published:** 2020-06-18

**Authors:** Hua Fan, Qiong Wu, Longping Peng, Du Li, Yidan Dong, Min Cao, Ping Liu, Xu Wang, Xudong Hu, Youhua Wang

**Affiliations:** ^1^ Cardiovascular Department, Longhua Hospital, Shanghai University of Traditional Chinese Medicine, Shanghai, China; ^2^ Department of Biology, School of Basic Medical Science, Shanghai University of Traditional Chinese Medicine, Shanghai, China; ^3^ Cancer Institute, Fudan University Shanghai Cancer Center, Shanghai, China

**Keywords:** *Phyllolobium chinense* Fisch flavonoids (PCFF), zebrafish, RAW264.7 macrophages, inflammatory response, nuclear factor-κB signaling pathway, inducible nitric oxide synthases

## Abstract

**Background:**

M1 macrophage plays an important role in inflammatory reaction. In this study, potential anti-inflammatory effect of *Phyllolobium chinense* Fisch flavonoids (PCFF) was assessed *via* Zebrafish acute inflammation model *in vivo* and LPS-induced pro-inflammatory M1 macrophage model *in vitro*.

**Methods:**

The quality control of *P. chinense* Fisch flavonoids (PCFF) was analyzed by HPLC. Anti-inflammatory effect of PCFF on the acute injured zebrafish was evaluated by the migration of fluorescence labeled macrophages and neutrophils, and the gene expression of inflammatory factors. In addition, the anti-inflammatory mechanism of PCFF was investigated by the related gene expression and related signaling pathway regulation of pro-inflammatory mediators in LPS-induced pro-inflammatory M1 RAW264.7 macrophage.

**Results:**

*P. chinense* Fisch flavonoids (PCFF) markedly suppressed macrophage and neutrophil migration and iNOS gene expression in acute injured zebrafish with tail-cutting. PCFF significantly inhibited NO overproduction and iNOS gene overexpression in LPS-sitimulated pro-inflammatory M1 RAW264.7 macrophages. What’s more, PCFF could evidently decrease p65 protein production, but had no effect on the production of P38, JNK and ERK1/2 proteins.

**Conclusion:**

*P. chinense* Fisch flavonoids (PCFF) have a remarkable inhibitory effect on the inflammatory response in acute injured zebrafish and LPS-stimulated M1 RAW264.7 macrophage. The pharmacological mechanism may be related to the regulation of NO overproduction and the inhibition of NF-κB/iNOS signaling pathway.

## Introduction

*Phyllolobium chinense* Fisch is the dry mature seeds of *Astragalus complanatus* R. Br. The main chemical constituents of *P. chinense* Fisch include flavonoids, triterpenoid saponins, sterols and so on. Among them, flavonoids are the main active ingredients of *P. chinense* Fisch. It has been showed that *P. chinense* Fisch flavonoids (PCFF) has many pharmacological effects, such as lowering lipid, bringing high blood pressure down, reducing blood sugar, inhibiting inflammation, improving immunity, etc. (Xu et al., 2018). However, the anti-inflammatory mechanism of PCFF is still unclear.

Acute inflammation is the immediate and early response of the body to the stimulation of inflammatory factors, such as pathogens, damaged cells or irritants. The main characteristic of acute inflammation is exudative change centered on vascular response (Hwang et al., 2016). Excessive inflammation leads to the release of an enormous number of inflammatory mediators, such as nitric oxide (NO), tumor necrosis factor-alpha (TNF-α) and interleukin-6 (IL-6), which cause cell necrosis and tissue damage, thus aggravate inflammation (Medzhitov, 2008; Chu et al., 2020). Nitric oxide (NO) is a small molecule compound that regulates neurotransmission and inflammatory response (Lee et al., 2017). In mammals, NO is produced by three different nitric oxide synthases (NOS), namely inducible NOS (iNOS), endothelial NOS (eNOS) and neuronal NOS (nNOS). iNOS gene is mainly expressed in activated macrophages (Janakiram and Rao, 2012). It is almost not expressed in normal cells, but only in inflammatory, hypoxic stress and other pathological conditions, which can stimulate the growth of inflammation and other diseases (Förstermann and Sessa, 2012; Xue et al., 2018). When iNOS gene expression is activated abnormally and NO is released in the wrong place or excessively produced in the body, it can increase inflammatory response by oxidative damage to healthy cells and tissues (Lange et al., 2009). Consequently, inhibiting the over-expression of iNOS gene and reducing the over-production of NO are important targets of anti-inflammatory drugs.

Lipopolysaccharide (LPS) binds to the toll like receptor 4 (TLR4) on the surface of macrophage membranes and participates in iNOS gene expression through activating intracellular mitogen-activated protein kinase (MAPK) or/and nuclear factor-κB (NF-κB) signaling pathways (Ci et al., 2010; Ha et al., 2011; Han et al., 2020; Shi et al., 2020).

The purpose of this study was to investigate the inhibitory effect of PCFF on inflammation and its related mechanisms. The inhibition of PCFF on inflammation was studied by observing the effects of PCFF on macrophage migration in acute injured zebrafish embryos and LPS-induced over-expression of inflammatory mediators in RAW264.7 macrophages. Meanwhile, the mechanism of PCFF inhibiting iNOS gene overexpression was studied by analyzing the changes of major genes and proteins in MAPK and NF-κB signaling pathways.

## Materials and Methods

### Equipment and Reagents

NMR Brucker AM-500, 600 NMR Spectrometer were bought from Germany. Mass spectrometer Finnigan LCQ Deca XP were bought from USA. High performance liquid meter: Chromatography instrument: Dalian Elite PDA230 high pressure constant current pump, DAD230 detector. Preparative high performance liquid chromatography was produced by Suzhou Huitong Chromatography Separation and Purification Company. Fluorescence microscope was bought from Olympus. Refrigerated centrifuge was bought from Eppendorf, Germany.

*P. chinense* Fisch was bought from Shanghai Hua Ji Pharmaceutical Co., Ltd. (Lot: 16113004), and its origin was Shaanxi Province, China. It was identified by Professor Wei Li, School of Chinese herbal medicine, Shanghai University of traditional Chinese medicine, according to the Chinese Pharmacopoeia 2015 edition, and the sample was kept in the Pharmacy Teaching and Research Office of Shanghai University of traditional Chinese medicine. Dulbecco’s Modified Eagle’s Medium (DMEM), fetal vobine serum (FBS), penicillin–streptomycin, trypsin-EDTA and lysis buffer were purchased from Thermo Fisher Scientific (Grand Island, NY, USA). Dimethyl sulfoxide (DMSO), lipopolysaccharide (LPS; from *Escherichia coli* strain) and phosphate buffered saline (PBS) were purchased from Sigma Chemical Co. (St. Louis, MO, USA). Cell Counting Kit-8 (CCK-8) was purchased from Dojindo Co. (Kumamoto, Japan). The TNF-α ELISA kits were purchased from R&D Systems, Inc. (St. Louis, MO, USA). Antibodies including GAPDH (#2118), iNOS (#2982), P38 (#8690), ERK1/2 (#4695), JNK (#9258), I-kappa B (#4812), p65(#3033), HRP-linked antibody (#7074) were purchased from Cell Signaling Technology (Beverly, MA, USA). Phosphatase and protease inhibitors (Sangon Biotech, China). BCA protein assay kit was purchased from Biomiga, USA. SDS-PAGE and ECL were purchased from Beyotime. PVDF membranes was purchased from Millipore, USA. Trizol was purchased from Roche. PrimeScriptTM RT reagent kit with gDNA Eraser and SYBR Premix Ex TaqTM II were purchased from Takara

### *P. chinense* Fisch Flavonoids (PCFF) Extract and Structural Analysis

Firstly, *P. chinense* Fisch (1 kg) were extracted for 2 h with 20 L boiling water. Secondly, the decoction was combined and concentrated to about 200 ml. Thirdly, the concentrated decoction was loaded on DM130 macroporous resin, which was then eluted by mobile phase composed of ethanol and water from 60 to 80%. Fourthly, the ethanol-eluted fraction was collected and concentrated to 200 ml, then ethanol was added to 1,000 ml. Finally, the precipitate in ethanol was removed and the upper clear liquid was concentrated to water-freeno water. The powder obtained was *P. chinense* Fisch flavonoids (PCFF). Half of the powder is stored at −20°C for reserve. The other is dissolved in 20 ml DMSO and repeated separation on a C18 column. Finally, 180 mg Compound 1 and 210 mg Compound 2 were obtained. The structures of compounds 1 and 2 were identified by ^1^H-NMR, ^13^C-NMR and ESI-MS used Brucker AM-500, 600 NMR Spectrometer and Finnigan LCQ Deca XP respectively.

### High Performance Liquid Chromatography of *P. chinense* Fisch Flavonoids (PCFF)

Chromatographic conditions: Welch Xtimate C18 (4.6 mm × 250 mm, 5 µm), detection wavelength 254 nm, column temperature 30°C, mobile phase of acetonitrile-0.1% phosphoric acid, gradient elution (0–10 min 10:90–20:80, 10–20 min 20:80:60, 20–25 min 60–60:40, 25–30 min 60:40–95:5, 30–35 min 95:5–10:90), flow rate: 1 ml/min.

Preparation of PCFF solution: 20 mg PCFF was weighed and put in 5 ml capacity bottle. After dissolving in methanol, PCFF was filtered by 0.45 μm microporous membrane as test solution. The determination was carried out according to the above chromatographic conditions.

Preparation of reference solution: Complanatuside A and complanatuside B were selected as reference substances. The solution containing complanatuside A and complanatuside B were 0.42 mg and 0.68 mg per ml respectively, which were to be used.

### Zebrafish Culture

The female (Cora1a: EGFP) and male (Lyz: DsRED) zebrafish were mating at a ratio of 2:2. The next morning, fish eggs were collected and cleaned with Embryo Medium, and incubated in 28.5°C incubators. Three days post-fertilization (3 dpf) zebrafish embryos were observed under fluorescence microscope, and both green fluorescence (macrophages’ mark) and orange fluorescence (neutrophils’ mark) Tg (Cora1a:EGFP/Lyz : DsRED) zebrafish were selected for subsequent experiments.

### Toxicity Test of Zebrafish

The 3 dpf Tg (Cora1a:EGFP/Lyz : DsRED) zebrafish were placed in 6-well plates containing Embryo Medium, 10 zebrafish in each well. Zebrafish were treated with PCFF (0, 15, 30, 50, 100 μg/ml) for 24 h in 28.5°C incubator, three wells per PCFF concentration. Then, the survival rate of zebrafish was observed.

### Acute Inflammatory Response in Zebrafish

The 3 dpf Tg (Cora1a: EGFP/Lyz: DsRED) zebrafish were placed in 6-well plates containing Embryo Medium, 10 zebrafish in each well. Zebrafish were divided into normal group, model group and PCFF (15, 30 μg/ml) treatment group, three wells per group. After anesthesia with 10× tricaine, the zebrafish tails of model and PCFF group were cut off. Then the zebrafish were treated without (normal, model) or with PCFF(15, 30 μg/ml) for 6 h in 28.5°C incubator. Then, the aggregation of macrophages and neutrophils in zebrafish were observed by fluorescence microscopy.

### Real-Time RT-PCR Analysis of Inflammation Related-Genes in Zebrafish

The 3 dpf Tg (Cora1a: EGFP/Lyz: DsRED) zebrafish were placed in 6-well plates containing Embryo Medium, 10 zebrafish in each well. Zebrafish were divided into normal group, model group and PCFF (15, 30 μg/ml) treatment group, three wells per group. After anesthesia with 10× tricaine, the zebrafish tails of control and PCFF group were cut off. Then the zebrafish were treated without (normal, model) or with PCFF (15, 30 μg/ml) for 6 h in 28.5°C incubator. After shaking zebrafish in PBST solution for 5 min and repeat for three times, the Trizol method was used to isolate total RNA from the zebrafish in each well. Then, 1 μg total RNA was reverse transcribed into cDNA, which was amplified by real-time PCR. The relative expression levels of target genes were quantified by housekeeping gene beta-actin and analyzed by 2^−ΔΔCT^ method.

The cycling conditions were as follows:

hold: 95°C for 30 s; cycling: 95°C for 5 s, 57°C for 30 s, 72°C for 30 s, 40 cycles; melt: 95°C for 15 s, 60°C for 60 s, 95°C for 15 s.

The sequences of primers (zebrafish) used for reverse transcription are listed below:

**Table:** 

iNOS	F: 5′-GGAGATGCAAGGTCAGCTTC-3′	R: 5′-GGCAAAGCTCAGTGACTTCC-3′
TNF-α	F: 5′-AAGGAGAGTTGCCTTTACCG-3′	R: 5′-ATTGCCCTGGGTCTTATGG-3′
IL-1β	F: 5′-TGGACTTCGCAGCACAAAATG-3′	R: 5′-CACTTCACGCTCTTGGATGA-3′
β-actin	F: 5′-CTTGGGTATGGAATCTTGCG-3′	R: 5′-AGCATTTGCGGTGGACGAT-3′

### Cell Culture

Murine RAW264.7 macrophages were purchased from the Shanghai Cell Bank of Chinese Academy of Sciences (China). RAW264.7 macrophages were cultured in DMEM supplemented with 10% FBS, 100 U/ml penicillin and 100 U/ml streptomycin. Cells were grown at 37°C in a humidified incubator with 5% CO_2_.

### Cell Viability

CCK-8 assay was used to determine the viability of RAW264.7 macrophages. Raw264.7 macrophages(1 × 10^5^/well) were grown in 96-well plates. After treating with PCFF (0, 12.5, 25, 50, 100, 200, 400, 800 μg/ml), three wells per PCFF concentration, for 24 h, macrophages were incubated with CCK-8 (10 μl/well) for 2–4 h, and then OD 450 nm was measured.

### Greiss Method

The level of nitrite (NO^2−^) in the cell culture supernatant, stably generated during NO reactions, was assessed to determine the amount of NO production. Firstly, Raw264.7 macrophages (1 × 10^5^/well) were grown in 96-well plates. Cells were untreated (control), or treated with LPS (1 μg/ml) and PCFF at different concentrations (0, 25, 50, 100, 200, 400 μg/ml), three wells per PCFF concentration, for 24 h. Secondly, Griess reagent was prepared by mixing 0.1% N-(1-naphthyl)ethylenediamine dihydrochloride (dissolved in ddH_2_O) and 1% sulfanilamide (dissolved in 5% H_3_PO_4_) in equal volume. Lastly, 100 μl of culture supernatant and 100 μl of Griess reagent were blended with a micropipette and set aside for 10 min at room temperature, then the absorbance at 540 nm was measured. A NaNO_2_ standard curve was used to calculate nitrite concentration.

### ELISA

The inhibitory effects of PCFF on pro-inflammatory cytokines produced by LPS-activated cells was detected by ELISA kit. Firstly, RAW264.7 macrophages (1 × 10^5^/well) were grown in 96-well plates. Cells were untreated (normal), or treated with only PCFF, only LPS (1 μg/ml), or both LPS and PCFF, 3 wells per group, for 24 h. Secondly, culture supernatants were collected and commercial ELISA kits were used to measure TNF-α concentrations.

### Real-Time RT-PCR Analysis of iNOS Gene in RAW264.7 Macrophages

Firstly, RAW264.7 macrophages (1 × 10^6^/well) were grown in 12-well plates. Cells were untreated (normal), or treated with only PCFF, only LPS (1 μg/ml), or both LPS and PCFF, three wells per group, for 24 h. Secondly, the Trizol method was used to isolate total RNA from the cells in each well. Thirdly, 1 μg total RNA was reverse transcribed into cDNA, which was amplified by real-time PCR. Finally, the 2^−ΔΔCT^ method was used to analyze gene expression and GAPDH mRNA served as an internal control to quantify the levels of target mRNAs relatively.

The cycling conditions were as follows:

hold: 95°C for 10 s; cycling: 95°C for 5 s, 60°C for 30 s, 40 cycles; melt: 65–95°C.

The sequences of primers (mice) used for reverse transcription are listed below:

**Table:** 

iNOS	F: 5′-GTTCTCAGCCCAACAATACAAGA-3′	R:5′-GTGGACGGGTCGATGTCAC-3′
GAPDH	F: 5′-TGACCTCAACTACATGGTCTACA-3′	R:5′-CTTCCCATTCTCGGCCTTG-3′

### Western Blotting

Firstly, RAW264.7 macrophages (1 × 10^6^/well) were grown in 12-well plates. Cells were untreated (normal), or treated with only PCFF, only LPS (1 μg/ml), or both LPS and PCFF, three wells per group, for 24 h. Secondly, cells were harvested on ice and washed once with ice-cold PBS. Lysis buffer with phosphatase and protease inhibitors (Sangon Biotech, China) was added to lyse the cells. After incubating on ice for 30 min, cell extracts were centrifuged at 14,463×*g* in a refrigerated centrifuge (5418R, Eppendorf, Germany) at 4°C for 10 min to collect cell total proteins, the amount of which was quantified using a BCA protein assay kit. Thirdly, SDS-PAGE (10%) was used to separate proteins, which were electro-transferred to PVDF membranes. Membranes were blocked with 5% (wt/vol) dried skimmed milk for 1 h, and incubated with various specific primary antibodies, namely, anti-GAPDH, anti-iNOS, anti-p65, anti-P38, anti-ERK1/2, anti-JNK, to probe corresponding target proteins. Bound antibodies were detected using peroxidase-conjugated secondary antibodies, and the amount of bound antibody was assessed by enhanced chemiluminescence (ECL). Finally, Relative levels of target proteins were obtained based on the optical density of electrophoresis bands with GAPDH serving as an internal control.

### Statistical Analysis

Data are presented as means (SD). Multiple comparisons were performed using the one-Way ANOVA test followed by Student–Newman–Keuls (SNK) and least significant difference (LSD) tests. P values <0.05 were considered to represent significant differences between means.

## Results

### Extraction, Separation and Structural Analysis of *P. chinense* Fisch flavonoids (PCFF)

**Figure 1:**
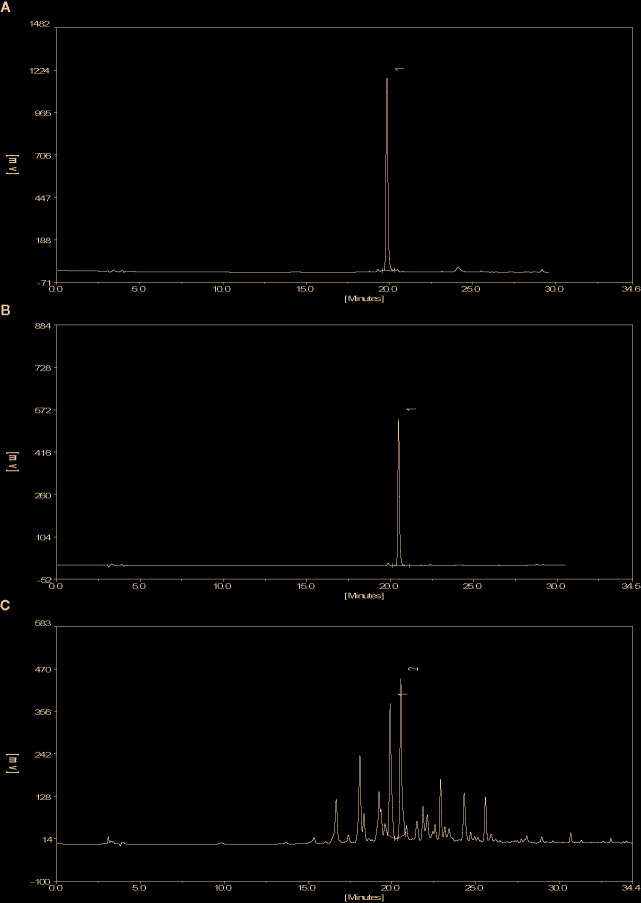
High Performance Liquid Chromatography of PCFF. **(A)** complanatosides B reference substance; **(B)** complanatosides A reference substance; **(C)** Sample.

The molecular structures of compounds 1 and 2 were identified by ^1^H-NMR, ^13^C-NMR and ESI-MS (**Supplementary S1** and **S2**). By comparing the chemical shifts with previous publications, we confirmed that the peak 1 is complanatosides B and the peak 2 is complanatosides A (**Figure 1**, **Table 1**). The content of Complanatoside A was 9.5% of total flavonoids of *P. chinense* Fisch, the content of Complanatoside B was 13.1%. It is suggested that the main constituents of *P. chinense* Fisch flavonoids (PCFF) are complanatosides A and complanatosides B, which can be used as quality control index for extracting PCFF.

**Table 1:** Compound of peak 1 and peak 2.

Peak	Compound	Retention Time (min)	structure
1	Complanatoside B	19.85	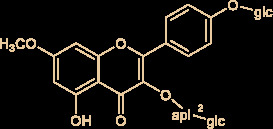
2	Complanatoside A	20.5	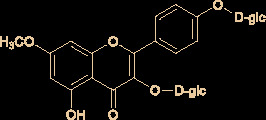

### Toxic Effect of PCFF on Zebrafish

**Figure 2:**
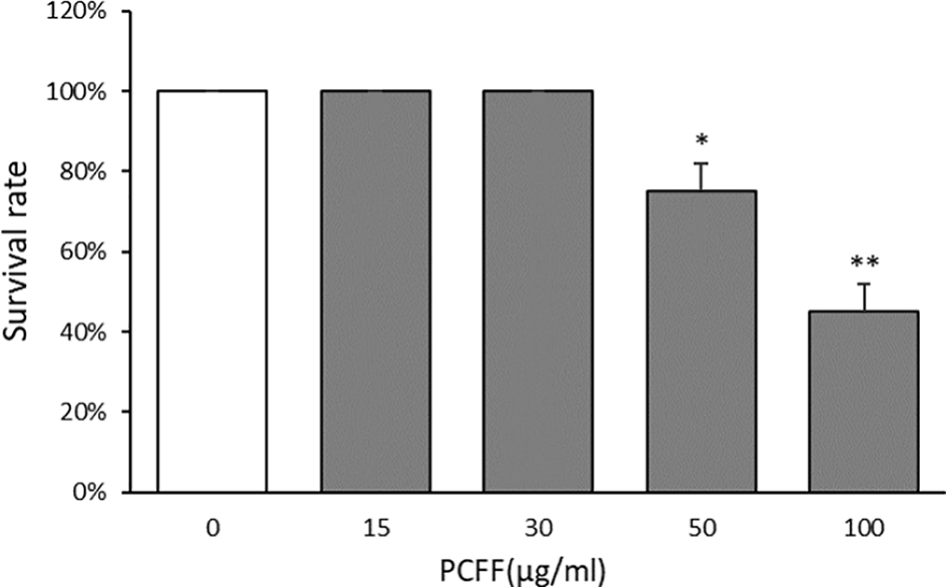
Survival rate of zebrafish. Each value indicates the mean ± SD from three independent experiments. Compared with normal group, ^*^P < 0.05, ^**^P < 0.01.

The three days post fertilization (3 dpf) Tg (Cora1a: EGFP/Lyz: DsRED) zebrafish were treated with PCFF (15, 30, 50, 100 μg/ml) for 24 h to observe the survival of zebrafish. The results showed that the morphology and heart rate of zebrafish were normal when the concentration of PCFF was below 30 μg/ml. Beginning with 50 μg/ml concentration, part of zebrafish showed deformity, body bending and cardiac arrest in a dose-dependent manner (**Figure 2**). Therefore, the concentration of PCFF at 15 and 30 μg/ml was selected for further study.

### Effect of PCFF on Inflammatory Cells Migration in Zebrafish

**Figure 3:**
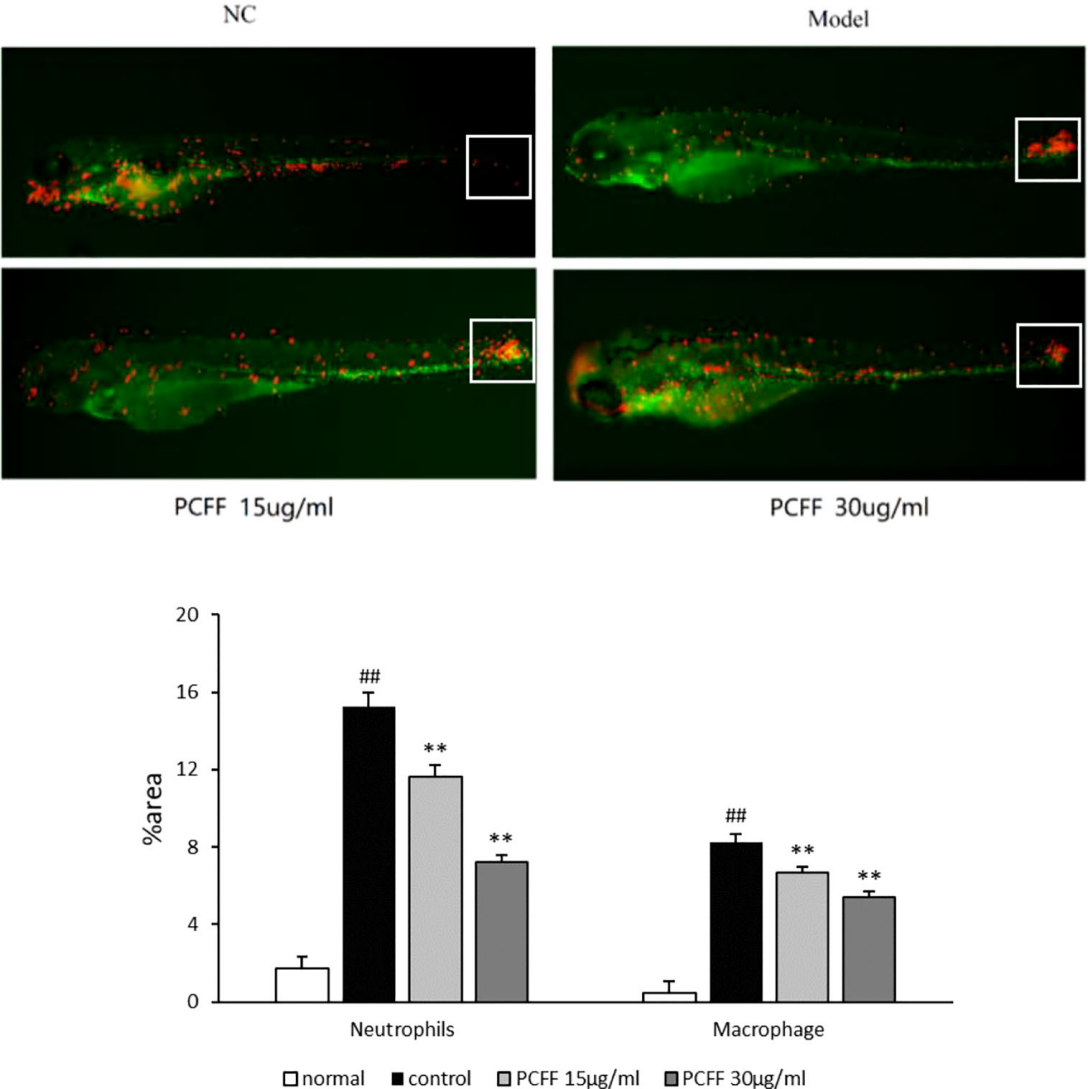
Inhibitory effect of PCFF on migration of zebrafish macrophages and neutrophils. Each value indicates the mean ± SD from three independent experiments. Compared with normal group, ^##^P < 0.01; compared with control group, ^**^P < 0.01. NC group untreated; Model group cut tail; PCFF group added different dose PCFF after tail cut.

The 3 dpf Tg (Cora1a: EGFP/Lyz: DsRED) zebrafish from Normal control group, Model group and PCFF (15, 30 μg/ml) treatment groups were photographed under fluorescence microscope. As showed in **Figure 3**, the distribution of neutrophils and macrophages in normal zebrafish is uniform; after tail cutting, a large number of neutrophils and macrophages gathered to the tail area to produce inflammatory reaction; and after treated with different concentrations of PCFF, the number of neutrophils and macrophages in the tail was decreased significantly in a concentration-dependent manner.

### Inhibitory Effect of PCFF on Inflammatory Factors in Zebrafish

**Figure 4:**
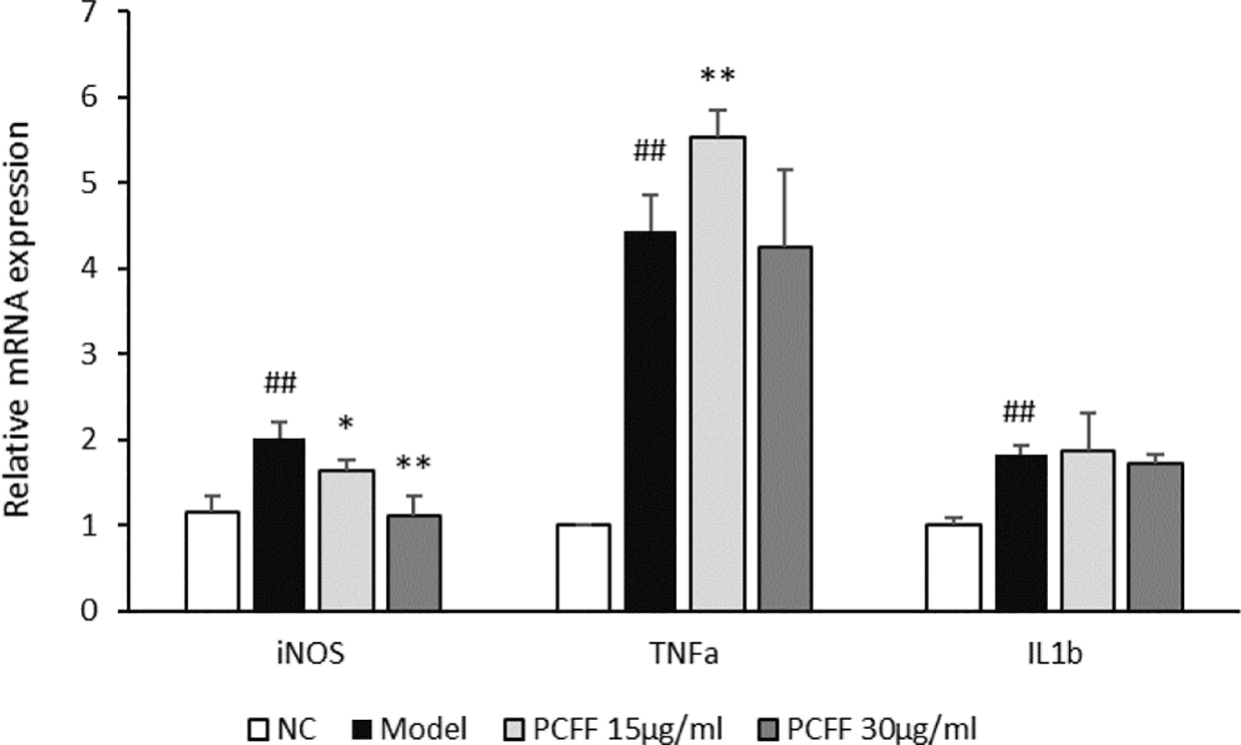
Inhibitory effect of PCFF on expression of iNOS、TNF-α and IL-1β mRNA. Each value indicates the mean ± SD from three independent experiments. Compared with normal group, ^##^P < 0.01; compared with control group, ^*^P < 0.05, ^**^P < 0.01. NC group untreated; Model group cut tail; PCFF group added different dose PCFF after cut tail.

The iNOS, TNF-α and IL-1β levels in 3 dpf Tg (Cora1a: EGFP/Lyz: DsRED) zebrafish were analyzed by RT-PCR. As showed in **Figure 4**, the expression of inflammatory genes, such as iNOS, TNF-α and IL-1β increased significantly after tail-cutting. PCFF can reduce the expression of iNOS mRNA, but has no inhibitory effect on TNF-α and IL-1β. Therefore, we try to further verify the anti-inflammatory mechanism of PCFF through *in vitro* experiments.

### Effects of PCFF on the Cytotoxicity of RAW264.7 Macrophages and the Production of NO

**Figure 5:**
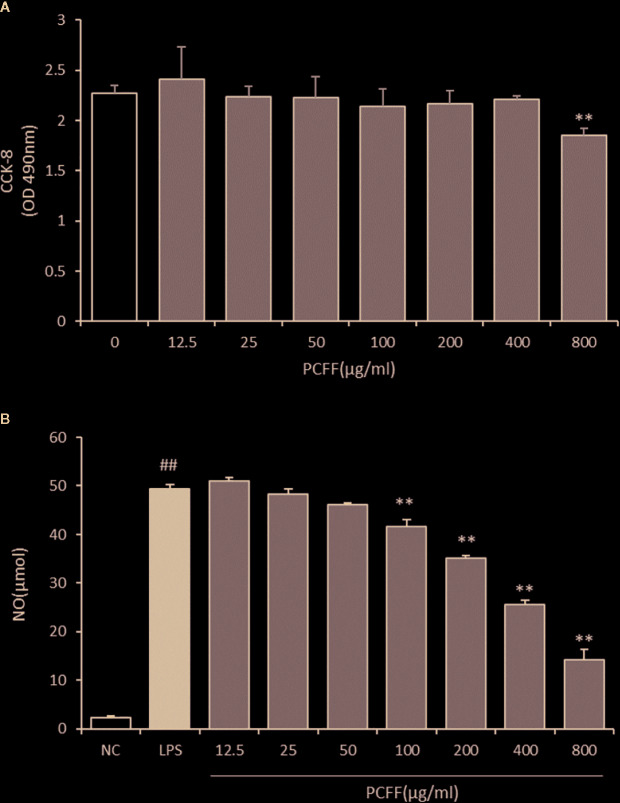
Cytotoxicity of PCFF on RAW264.7 cells and effects of PCFF on LPS-induced NO production in RAW264.7 cells. **(A)** Each value indicates the mean ± SD from three independent experiments. Compared with untreated group, ^**^P <0.01; **(B)** Compared with NC group, ^##^P < 0.01; compared with LPS group, ^**^P <0.01.

In zebrafish experiments, we found that PCFF can reduce the activation of macrophages and the inflammatory response of the body. Therefore, we tried to elucidate the anti-inflammatory mechanism of PCFF *in vitro*. First, we used CCK-8 to detect the survival rate of RAW264.7 macrophages at different concentrations of PCFF (0–800 μg/ml). The results showed that PCFF had no cytotoxic effect on RAW264.7 macrophages when PCFF was less than 400 µg/ml (**Figure 5A**). We further examined the effect of PCFF on NO production in LPS-induced M1 macrophages. The results showed that PCFF inhibited NO production in a dose-dependence manner, and the highest inhibition rate of NO production was 65.2% at 400 μg/ml (**Figure 5B**). Therefore, the concentration of PCFF at 400 μg/ml was selected for subsequent experiments.

### Inhibition Effects of PCFF on iNOS Gene Expression in LPS-Stimulated M1 Pro-Inflammation RAW264.7 Macrophage

**Figure 6:**
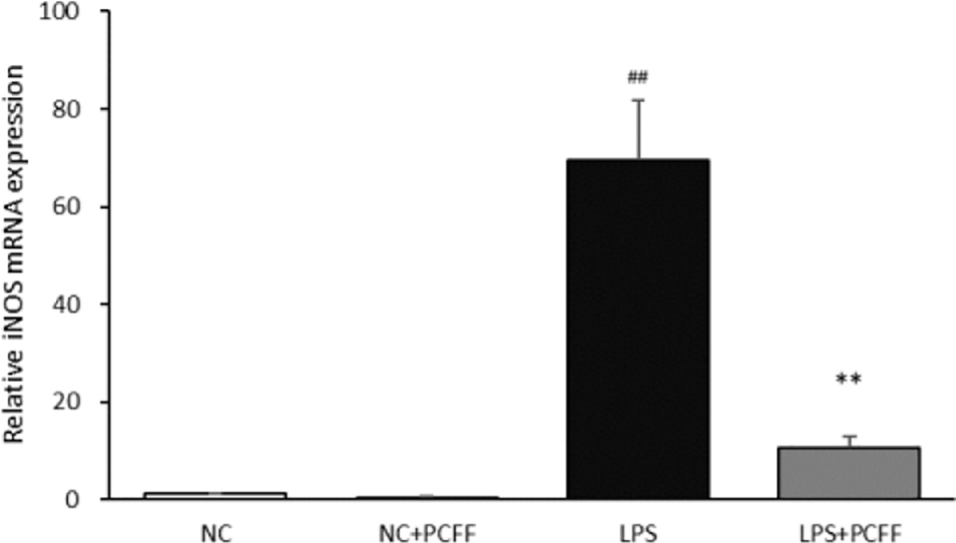
Effect of PCFF on LPS-induced inflammatory factor iNOS gene in RAW264.7 cells. Each value indicates the mean ± SD from three independent experiments. Compared with NC group, ^##^P <0.01; compared with LPS group, ^**^P <0.01.

Inducible nitric oxide synthase (iNOS) is the main catalytic enzyme for NO production during macrophage inflammation. The results show that, PCFF can significantly reduce iNOS gene transcription (**Figure 6**).

### Inhibitory Effect of PCFF on the Production of Inflammatory Factor TNF-α in LPS-Stimulated M1 Pro-Inflammation RAW264.7 Macrophage

**Figure 7:**
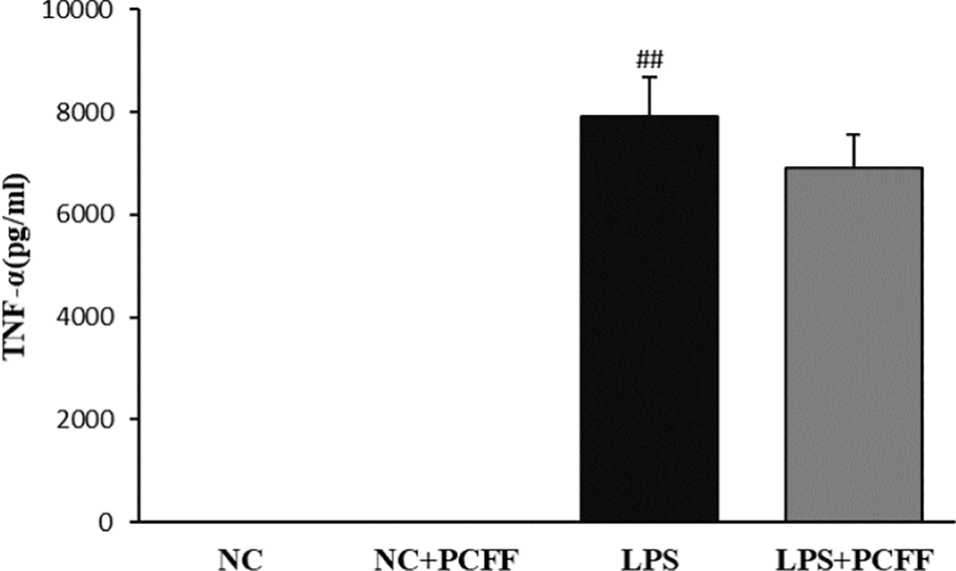
Effects of PCFF on TNF-alpha secretion in LPS-induced RAW264.7 cells. Each value indicates the mean ± SD from three independent experiments. Compared with NC group, ^##^P < 0.01.

TNF-α is also an important inflammatory mediator and marker of M1 polarization of LPS-stimulated macrophages. The results showed that PCFF did not significantly inhibit TNF-α production in RAW264.7 macrophages induced by LPS (**Figure 7**).

From here we see that PCFF may play an anti-inflammatory role by selectively inhibiting NO and iNOS gene production. Therefore, we attempt to elucidate the molecular mechanism of PCFF on inhibiting iNOS production in our follow-up studies.

### PCFF Regulates iNOS Protein Expression Through NF-κB Signaling Pathway

**Figure 8:**
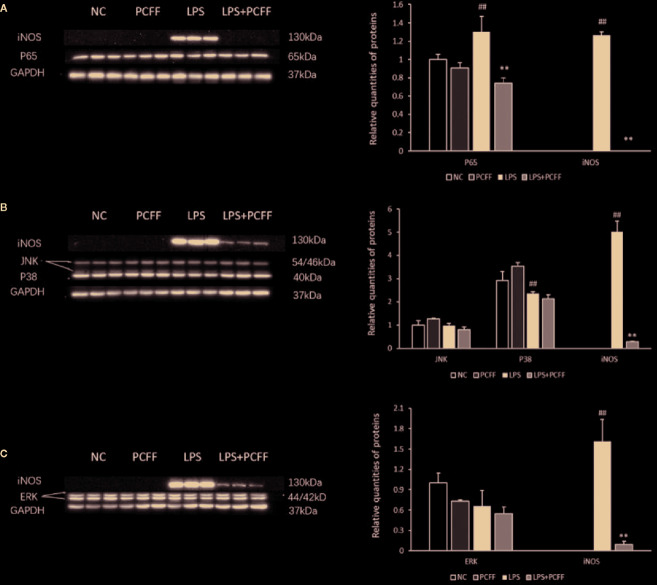
Effects of PCFF on NF-κB and MAPK signaling pathway proteins in LPS-stimulated RAW264.7 macrophages. **(A)** Effect of PCFF on p65 and iNOS proteins; **(B)** Effect of PCFF on JNK, P38 and iNOS proteins; **(C)** Effect of PCFF on ERK1/2 and iNOS proteins. Each value indicates the mean ± SD from three independent experiments. Compared with NC group, ^##^P < 0.01; compared with LPS group, ^**^P < 0.01.

MAPK and NF-κB signaling pathway are the main signaling pathways regulating iNOS gene expression. The results showed that LPS significantly increased the iNOS gene expression by stimulating p65 protein production in RAW264.7 macrophages, while PCFF significantly inhibited the excessive production of p65 (**Figure 8A**). PCFF had no significant effect on the production of P38, JNK and ERK1/2 (**Figures 8B, C**). These results suggested that PCFF could reduce iNOS gene overexpression by inhibiting the activation of NF-κB signaling pathway.

## Discussion

Inflammation is a basic defense response of the body against harmful stimuli, such as pathogens, damaged cells and wounds. However, this response generates pathological consequences that lead to inflammatory tissue damage, metaplasia, and a shift in homeostatic set points (Medzhitov, 2008). The zebrafish (*Danio rerio*), as a model organism, has been widely used in scientific research, including immunology (Rosowski, 2020; Sommer et al., 2020). Zebrafish larvae are suitable for investigating the innate immune response in vertebrates because their adaptive immune system matures in morphology and function 4–6 weeks after fertilization, while only innate immune response exists in larvae. In addition, transparency in zebrafish early life stages allows for useful real-time visualization (Novoa and Figueras, 2012). In this study, zebrafish embryos were traced with Tg (Cora1a: EGFP/Lyz: DsRED) double-labeled transgene line to detect macrophages and neutrophils. Macrophages were green fluorescence-labeled and neutrophils were red-green fluorescence-labeled. 3 dpf (days post fertilization) zebrafish were cut tail to induce inflammatory response. The migration of fluorescent labeled macrophages, neutrophils was observed and the inflammation related gene expression was detacted. Our results showed that *P. chinense* Fisch flavonoids (PCFF) could significantly decrease macrophages and neutrophils migration and alleviate iNOS gene expression, suggesting that PCFF could significantly inhibit the inflammatory response *in vivo* and the iNOS gene may be the key target of PCFF on the inhibition of macrophage activation.

Macrophages are innate immune cells widely distributed in the body and play an important role in inflammatory response (Yu et al., 2017; Lee et al., 2020). Macrophages can be divided into M1 proinflammatory type and M2 anti-inflammatory type according to the phenotype and function (Wynn et al., 2013; Orecchioni et al., 2019). Macrophages will polarize to M1 proinflammatory type after stimulated by lipopolysaccharide (LPS). Gene expression of inducible nitric oxide synthase (iNOS) was enhanced and iNOS increased significantly, which catalyzed L-arginine decomposition to produce excessive nitric oxide (NO). Nitrite peroxide produced by excessive NO and O^2−^ leads to cell death, extensive tissue damage and pathological changes through strong peroxidation. Therefore, the regulation of iNOS gene expression and activity is the most direct and key target in the treatment of inflammatory diseases involving NO. Our data demonstrated that *P. chinense* Fisch flavonoids (PCFF) could significantly reduce the excessive production of NO but not TNF-alpha in LPS-activated RAW264.7 macrophages, suggesting that PCFF suppressed inflammation by inhibiting the production of NO. Further study dicovered that PCFF inhibited NO overproduction by significantly inhibiting iNOS gene expression.

The promoters of iNOS gene include identifying sites such as NF-κB, AP-1, STAT-1a and so on. Stimulated by LPS, many pathways such as NF-κB signaling pathway, MAPK signaling pathway and protein kinase C signaling pathway can be activated. After cascade amplification, iNOS gene expression is increased, resulting in excessive NO production (Ji and Vina, 2007; Li et al., 2009; Dai et al., 2011). MAPK signaling pathway is composed of extracellular signal-regulated protein kinase (ERK), c-Jun N-terminal kinase and P38 MAPK (Lee et al., 2019). NF-κB is an important downstream target of the MAPK signaling pathway. The transfer of NF-κB dimer (mainly p65:p50 dimer) from cytoplasm to nucleus and will promote iNOS gene expression (Luo et al., 2020; Zhou et al., 2020). *P. chinense* Fisch flavonoids (PCFF) could significantly inhibit the production of total p65 protein in LPS-stimulated M1 RAW264.7 macrophage, but had no effects on total P38, JNK and ERK1/2 protein. Because PCFF has a significant inhibition on the production of total p65 protein, so we did not further detect the phosphorylated p65. These results suggested that PCFF could inhibit iNOS gene overexpression by inhibiting the NF-κB signaling pathway, thus reducing NO overproduction and inhibiting the M1 pro-inflammatory polarization of LPS-stimulated RAW264.7 macrophage.

In conclusion, *P. chinense* Fisch flavonoids (PCFF) can reduce macrophage activation in zebrafish with acute injury, and alleviate inflammation by inhibiting iNOS gene expression. The inhibitory effect of PCFF on M1 pro-inflammatory polarization of macrophage *in vitro* can be achieved by regulating NF-κB signaling pathway to inhibit the activity of iNOS and then down-regulate the expression of pro-inflammatory factor NO.

## Data Availability Statement

The raw data supporting the conclusions of this article will be made available by the authors, without undue reservation, to any qualified researcher.

## Author Contributions

All authors participated in the conception, design, interpretation, and elaboration of the findings of the study, as well as in drafting and revising the final version. In particular, HF, XW, LP, and DL maintained zebrafish cultures and carried out drug concentration screening. HF and YD carried out fluorescence detection of zebrafish. HF, QW, and PL determined zebrafish protein. and MC maintained cell cultures and carried out cell survival experiments. HF, QW, and DL determined RNA and protein of cells. QW and PL tested NO and TNF-α production. YW, XH, HF, and QW participated in the elaboration of the findings of the study, drafting and revising the final version. Supervision: XH and YW. All authors contributed to the article and approved the submitted version.

## Funding

This research was funded by National Natural Science Foundation of China Municipality (No. 81873264), the 2018–2020 Three-year Action Plan for Traditional Chinese Medicine Further Development in Shanghai (No. ZY (2018–2020) CCCX–2002-04); Budget Project of Shanghai University of Traditional Chinese Medicine (Natural Science) (No. 2019LK004); Longhua Medical Scholar Project (No. LYTD-86).

## Conflict of Interest

The authors declare that the research was conducted in the absence of any commercial or financial relationships that could be construed as a potential conflict of interest.

## Abbreviations

PCFF, *Phyllolobium chinense* Fisch flavonoids; NO, nitric oxide; TNF-α, tumor necrosis factor-alpha; LPS, lipopolysaccharide; MAPK, mitogen-activated protein kinase; NF-κB, nuclear factor-κB.
