# Supplementary figures and images for: Phyllolobium chinense Fisch Flavonoids (PCFF) Suppresses the M1 Polarization of LPS-Stimulated RAW264.7 Macrophages by Inhibiting NF-κB/iNOS Signaling Pathway
Source: Front Pharmacol. 2020 Jun 18;11:864. doi: 10.3389/fphar.2020.00864 (PMC7314944; doi:10.3389/fphar.2020.00864)

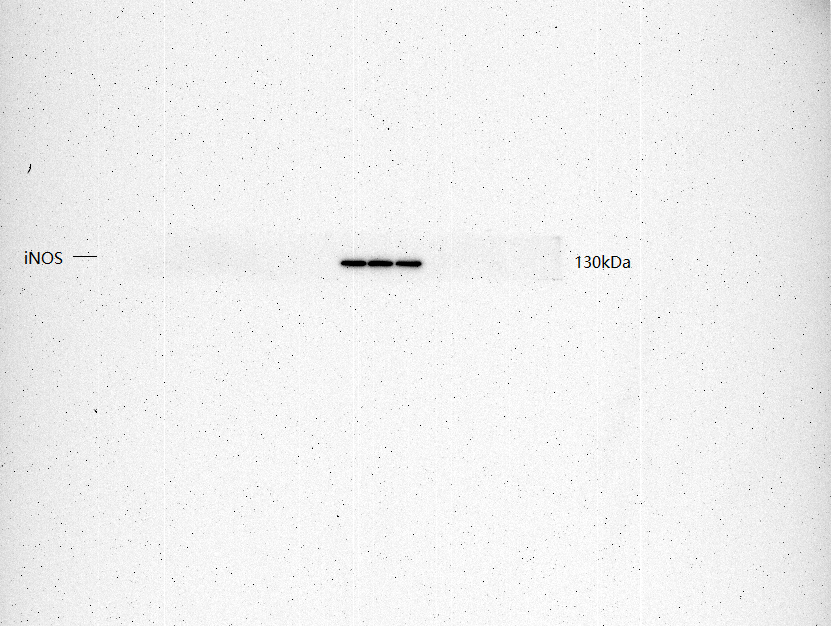

Supplement: Supplementary file 1 [file DataSheet_1.zip › (For Fig8A) Picture A-1. original image of iNOS band.png]

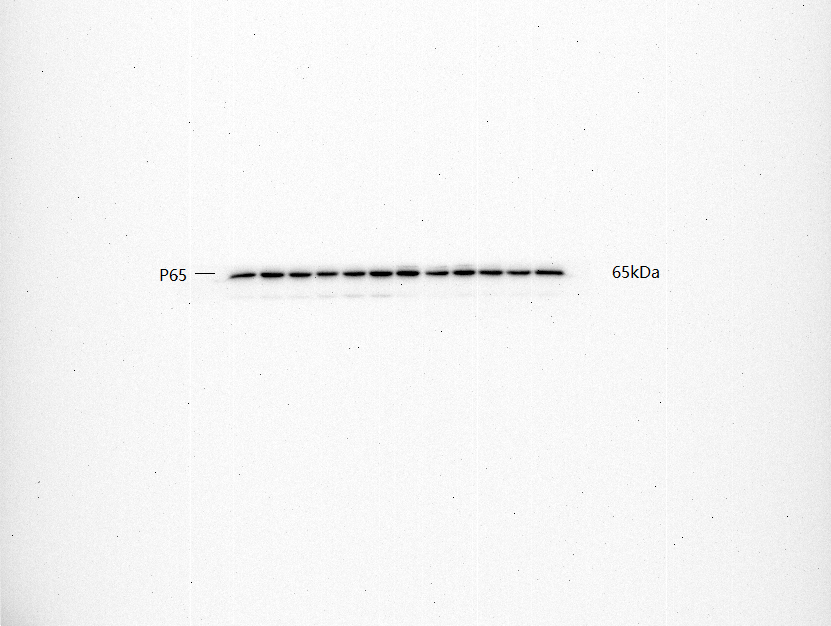

Supplement: Supplementary file 1 [file DataSheet_1.zip › (For Fig8A) Picture A-2. original image of P65 band.png]

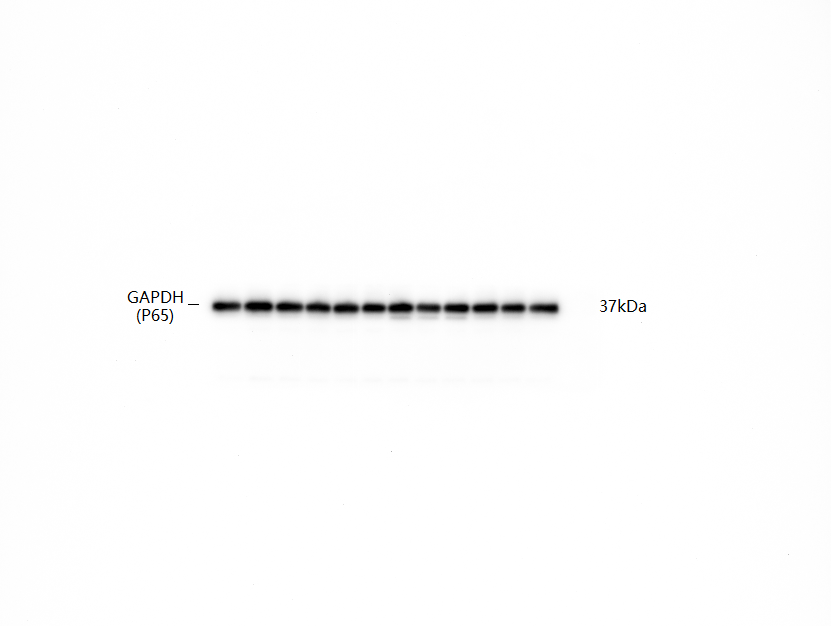

Supplement: Supplementary file 1 [file DataSheet_1.zip › (For Fig8A) Picture A-3. original image of GAPDH band.png]

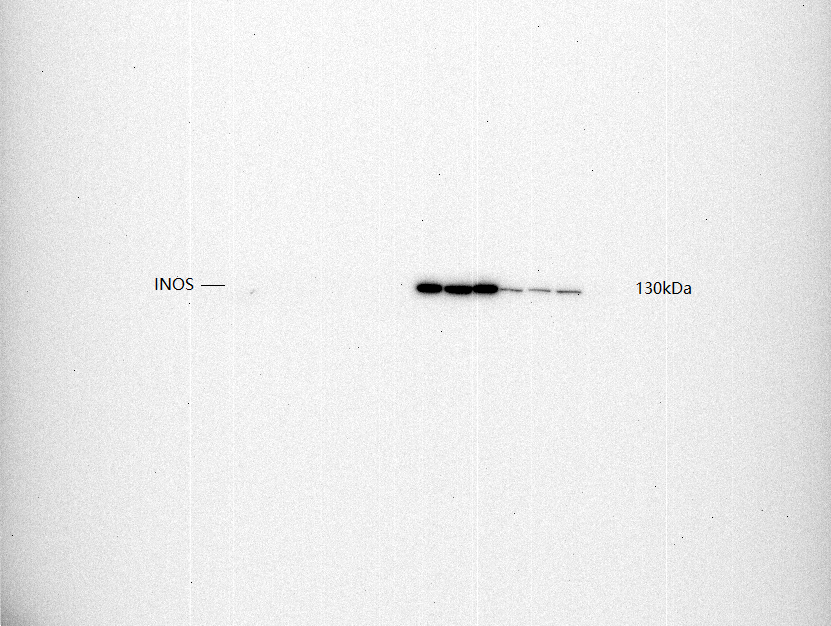

Supplement: Supplementary file 1 [file DataSheet_1.zip › (For Fig8B) Picture B-1. original image of iNOS band.png]

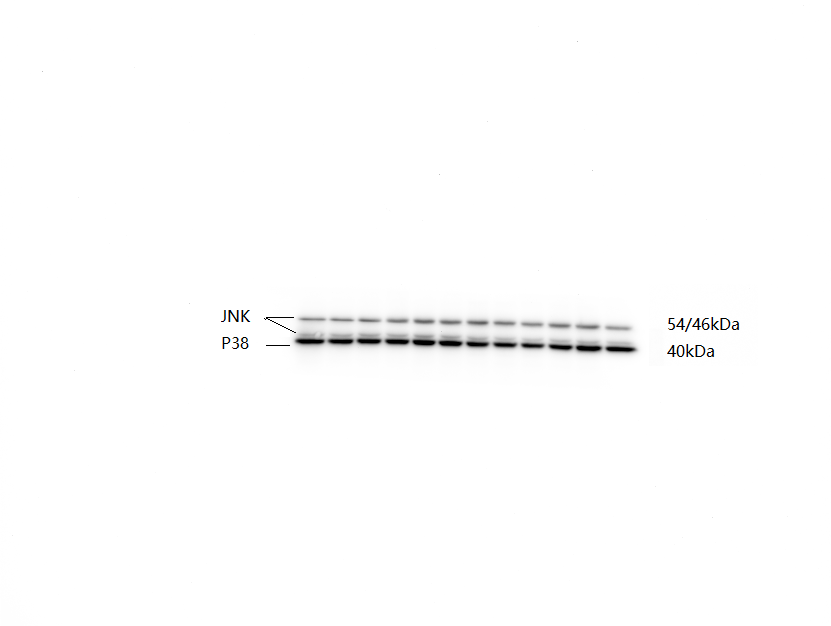

Supplement: Supplementary file 1 [file DataSheet_1.zip › (For Fig8B) Picture B-2. original image of JNK-P38 bands.png]

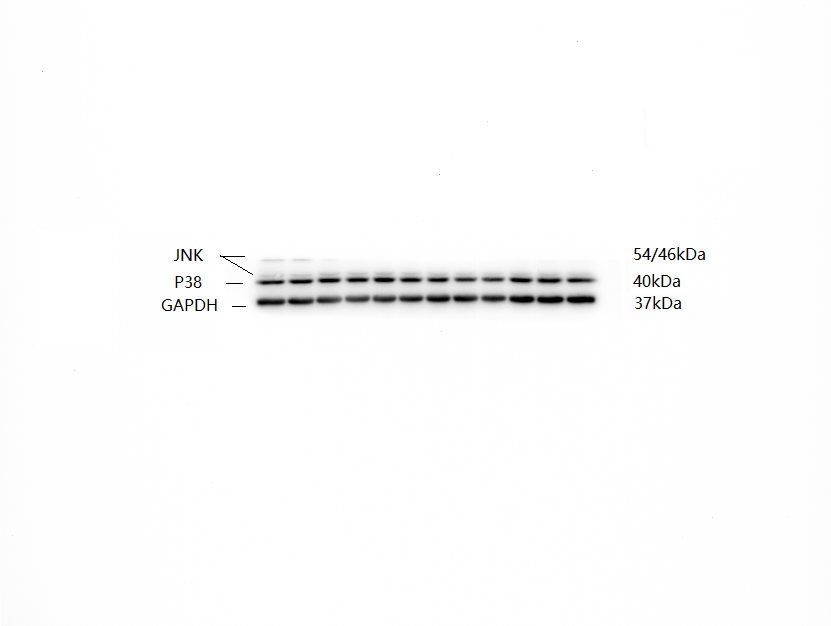

Supplement: Supplementary file 1 [file DataSheet_1.zip › (For Fig8B) Picture B-3. original image of GAPDH band.png]

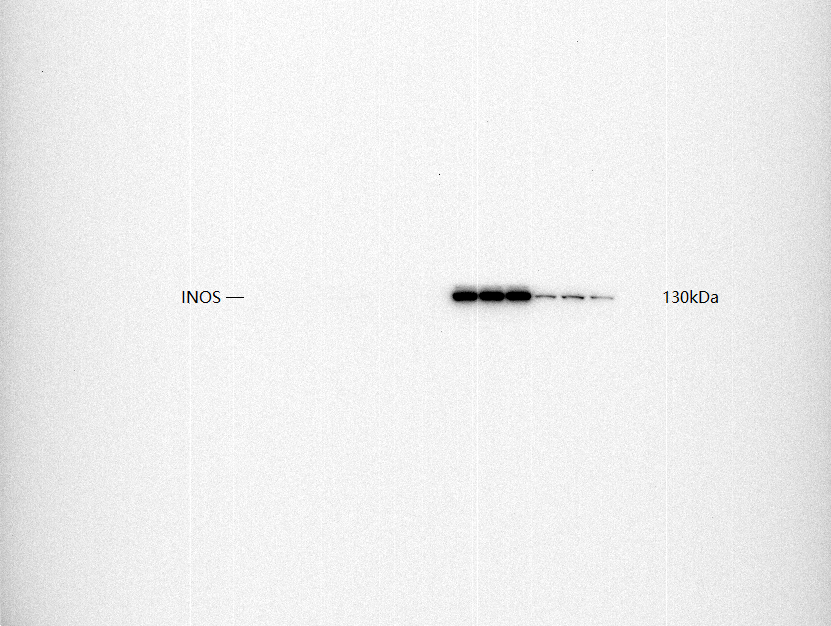

Supplement: Supplementary file 1 [file DataSheet_1.zip › (For Fig8C) Picture C-1. original image of iNOS band.png]

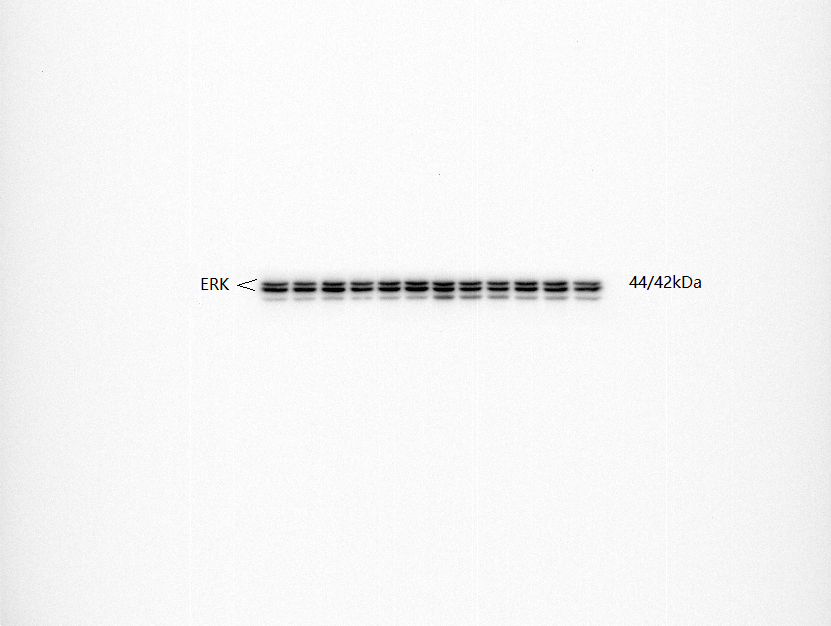

Supplement: Supplementary file 1 [file DataSheet_1.zip › (For Fig8C) Picture C-2. original image of ERK band.png]

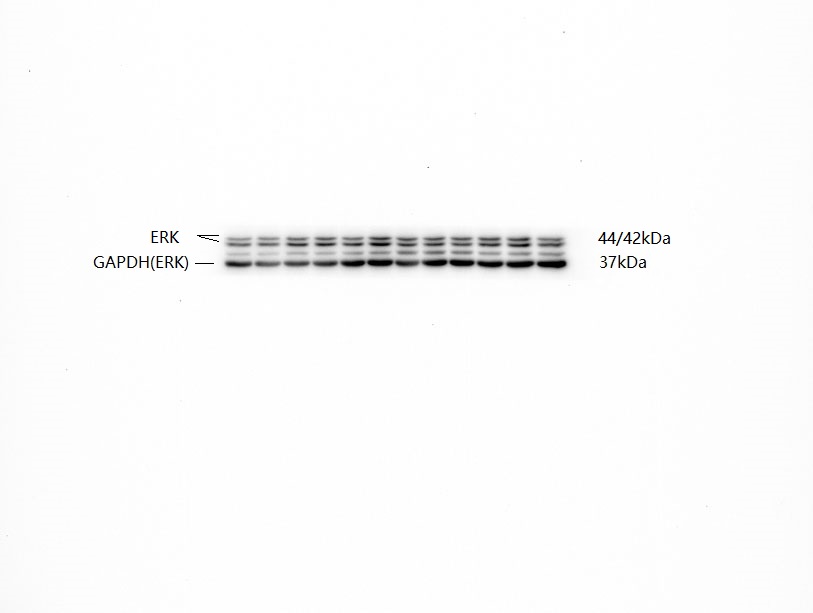

Supplement: Supplementary file 1 [file DataSheet_1.zip › (For Fig8C) Picture C-3. original image of GAPDH band.jpg]

# Supplementary Material

## S1


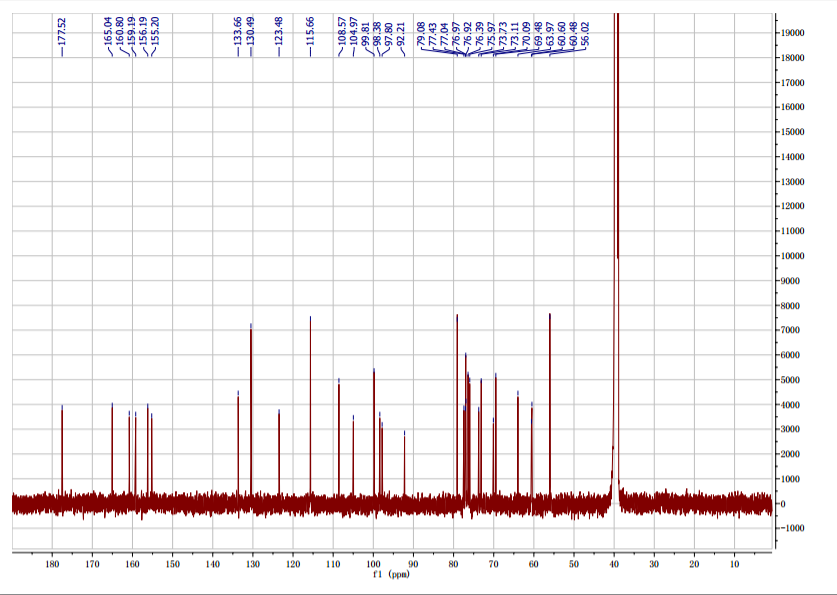


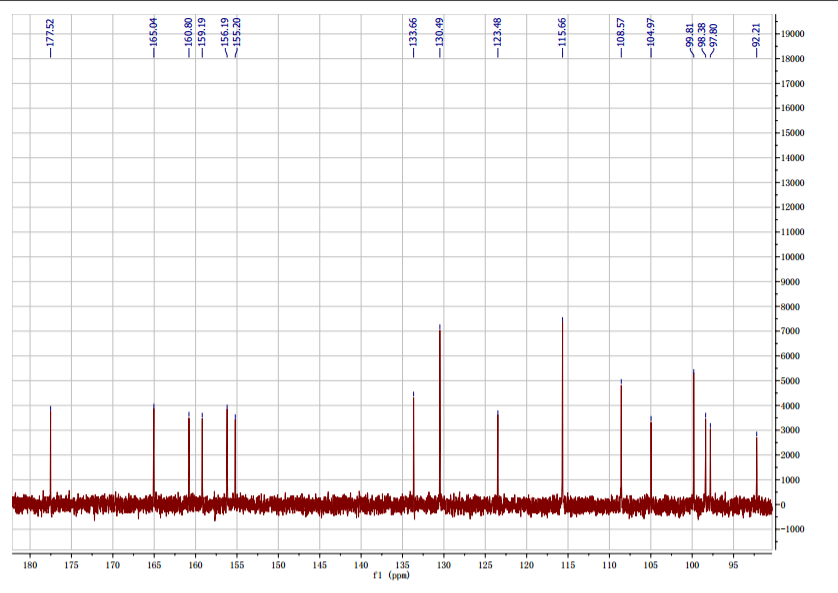


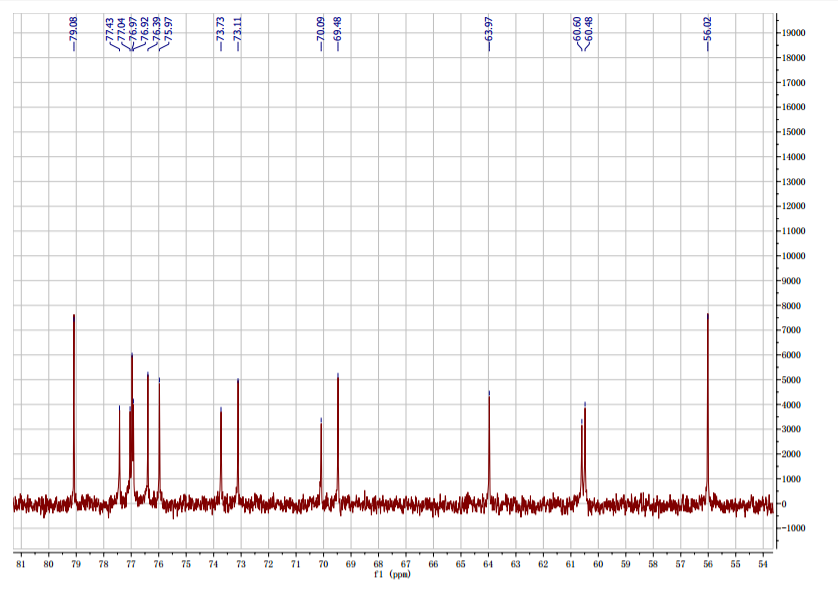

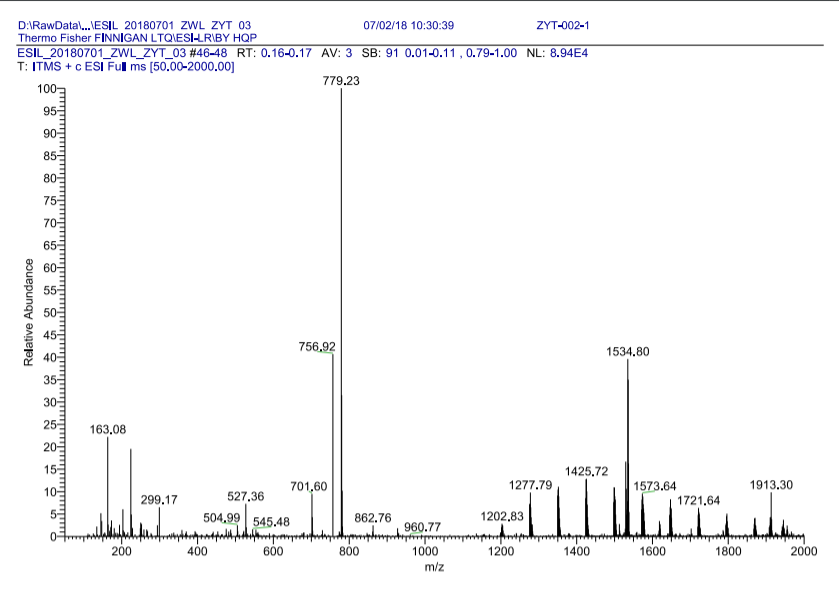

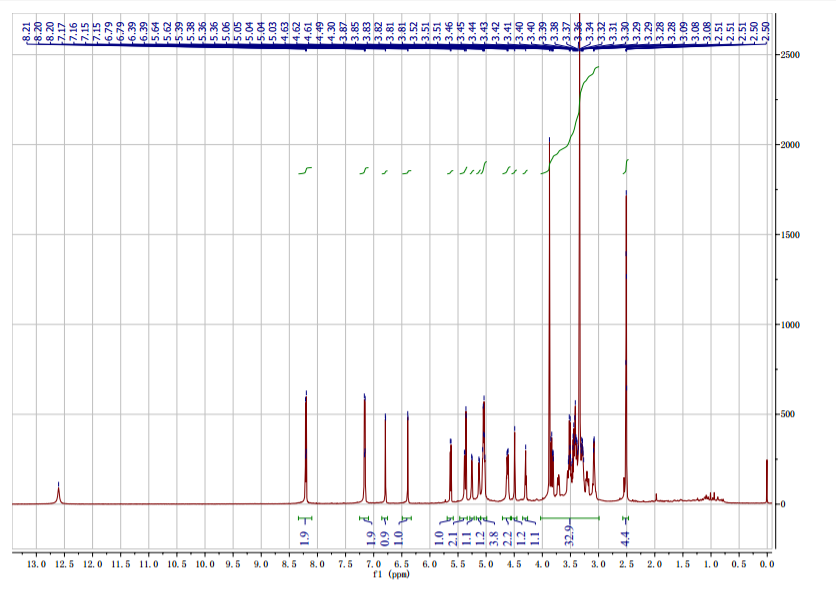

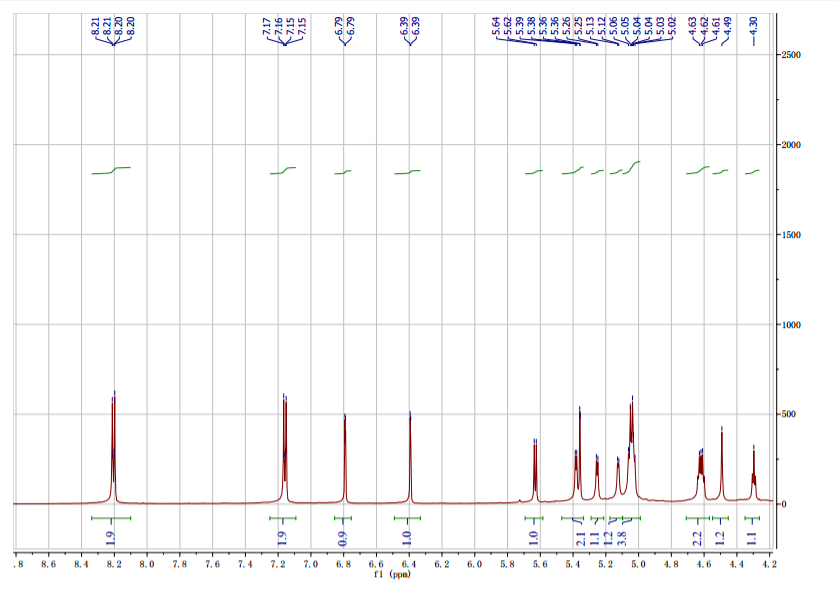

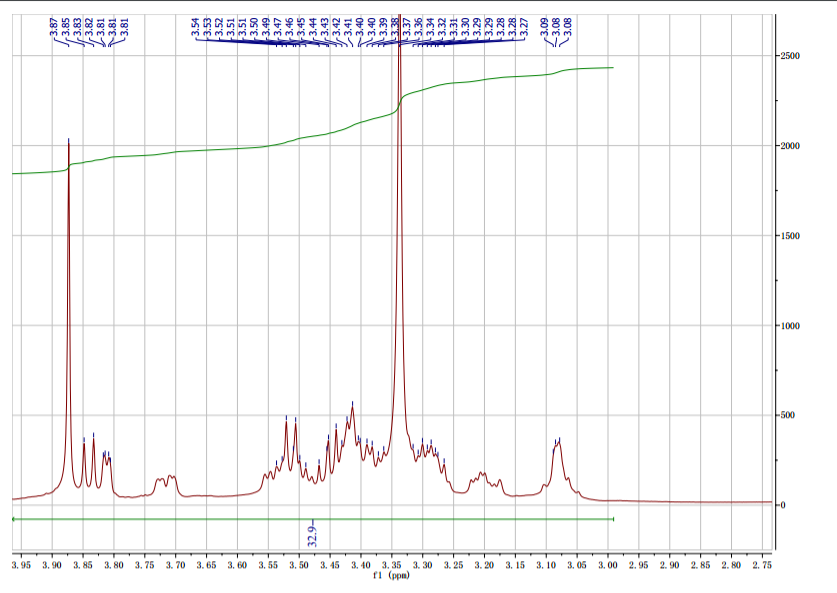


## S2


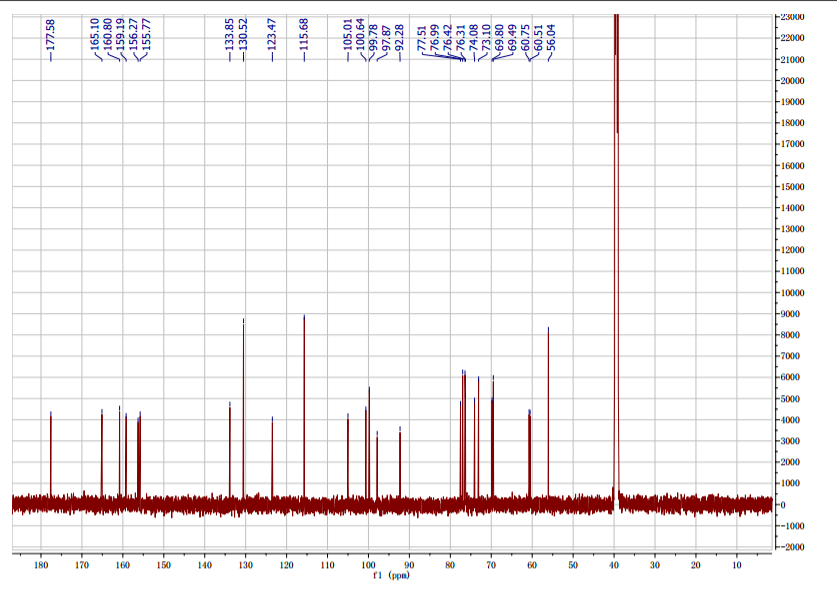

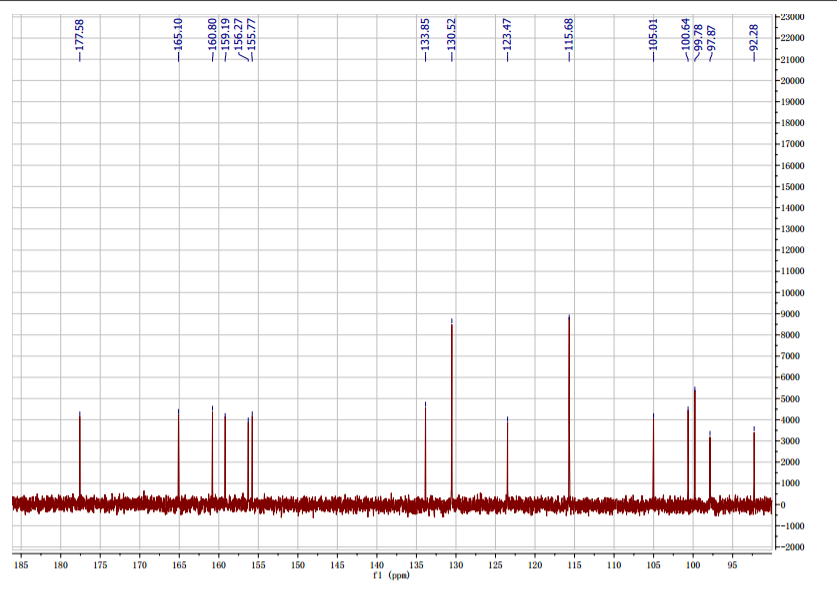

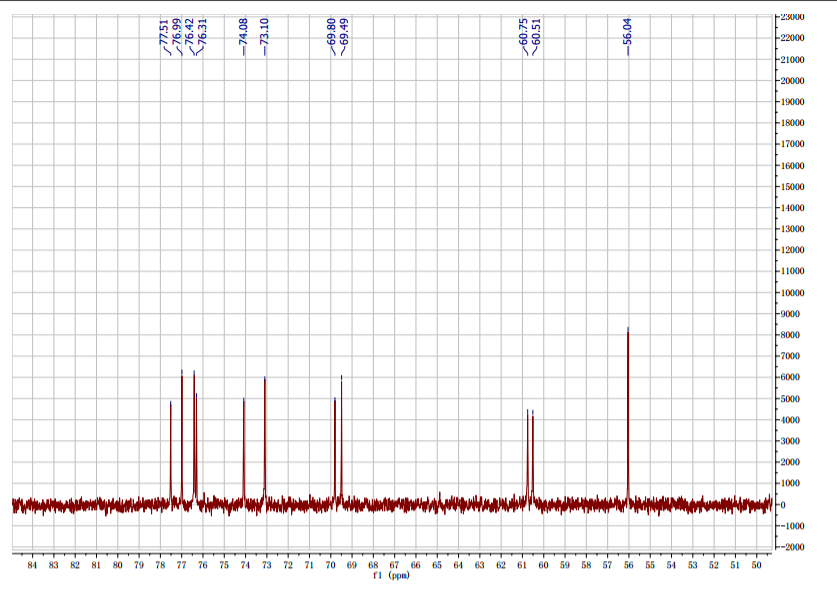

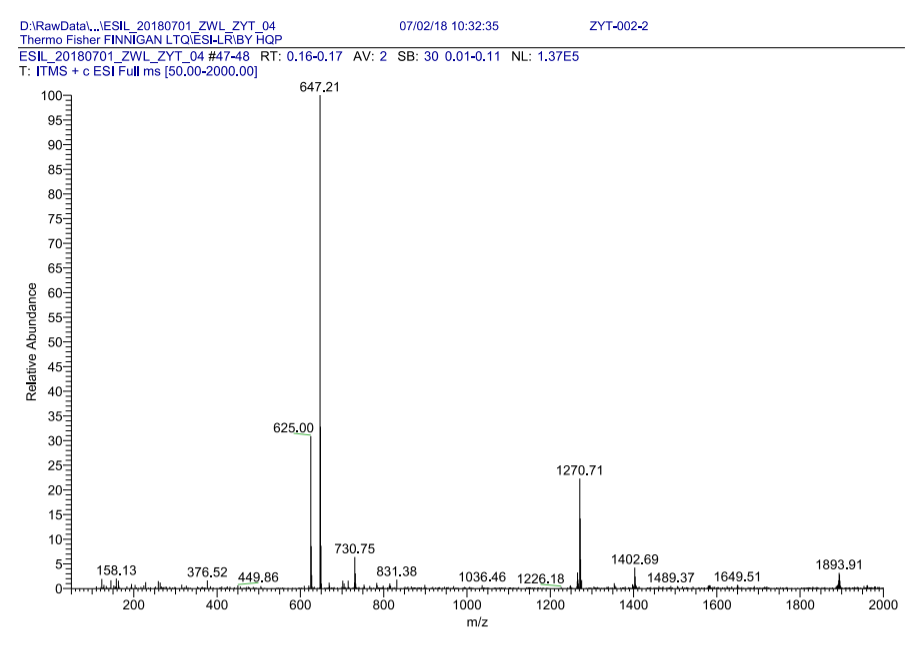

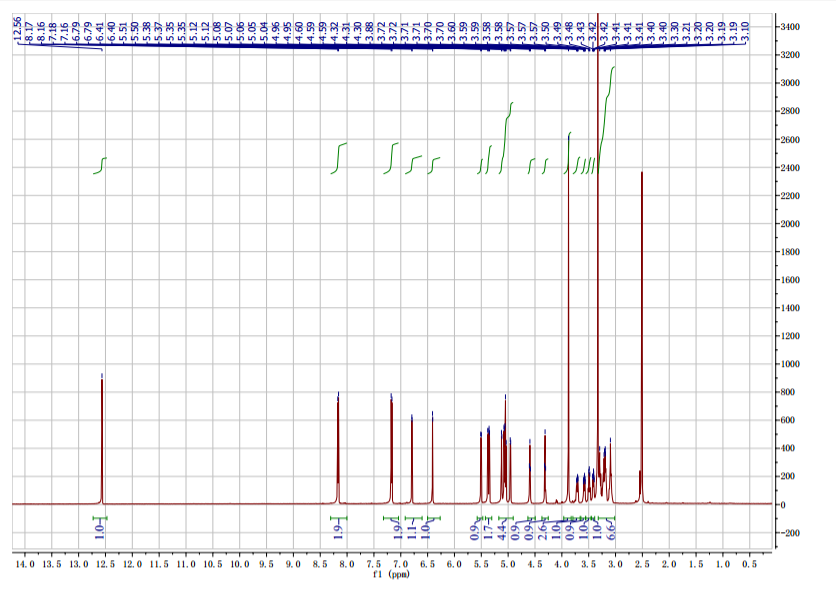

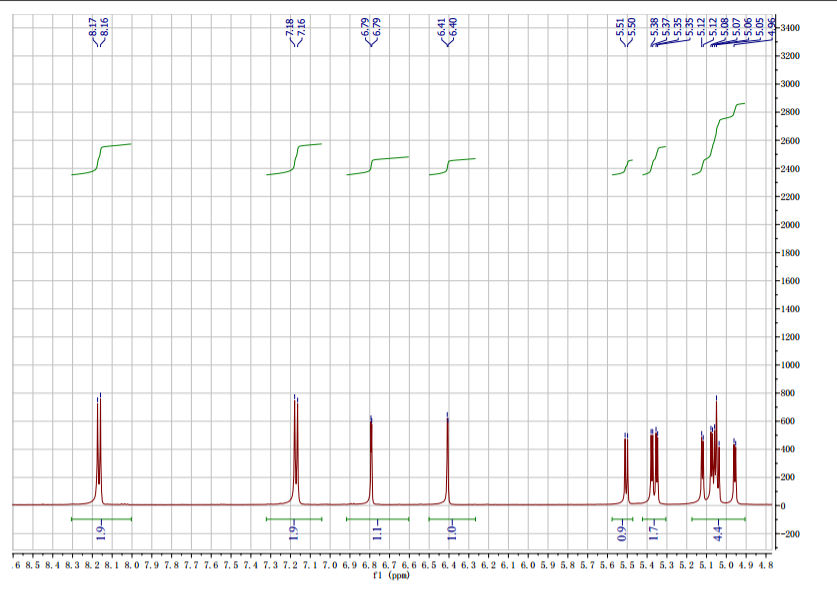

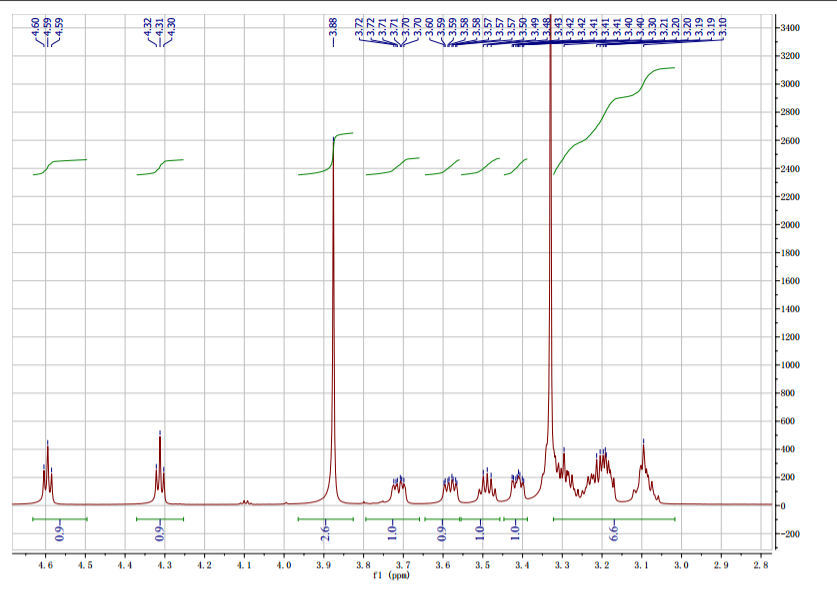

Supplement: Supplementary file 3 [file DataSheet_3.docx]
