# Supplementary material for: Phyllolobium chinense Fisch Flavonoids (PCFF) Suppresses the M1 Polarization of LPS-Stimulated RAW264.7 Macrophages by Inhibiting NF-κB/iNOS Signaling Pathway
Source: Front Pharmacol. 2020 Jun 18;11:864. doi: 10.3389/fphar.2020.00864 (PMC7314944; doi:10.3389/fphar.2020.00864)
Supplement: Supplementary file 2 [file DataSheet_2.docx]

**Supplementary Files**

**1.Chemical components identified in ACF（The total flavone of Astragali complanati semen）**

As shown in the HPLC chromatograms in Fig. 1, HPLC analysis successfully separated chemicals components in ACF. According to the spectrum, it can be judged that the peak1 and peak 2 accounts for the most in ACF. Then We Separated and identified the structure of peak 1 and 2 by 1H-NMR, 13C-NMR and ESI-MS and according to the research of Cui B L (Chem Pharm Bull, 1993) and Xu, M.Y(Zhongguo Zhong Yao Za Zhi. 2018), we find that the peak 1 is complanatosides B and the peak 2 is complanatosides A. The content of Complanatoside A in ACF was 9.5%, the content of Complanatoside B was 13.1%.

| 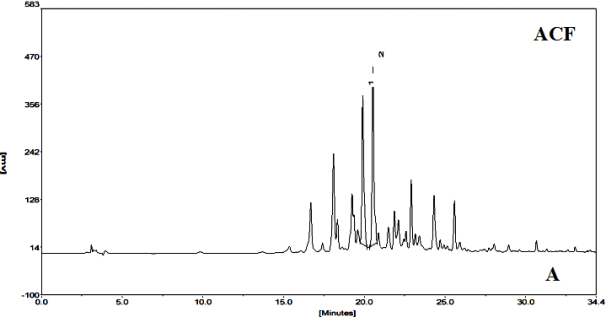 | |
| --- | --- |
| 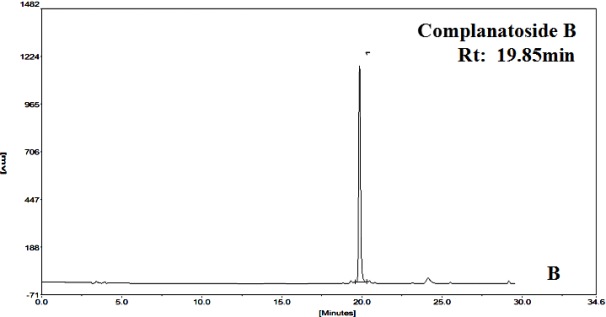 | 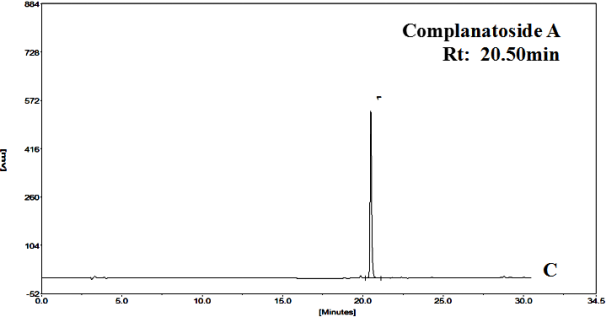 |

**Figure 1.** **Representative HPLC chromatograms of ACF.** A: ACF; B: Complanatoside B; C:Complanatoside A. Retention times (Rt) were shown in figure.

**Table 1.** compound of peak 1 and peak 2.

| Peak | compound | Retention Time (min) | structure |
| --- | --- | --- | --- |
| 1 | Complanatoside B | 19.85 | **** |
| 2 | Complanatoside A | 20.5 | **** |

**2.The extraction and identification identifications of chemical components in ACF**

We identified the molecular structures of compound 1 and 2 by 1H-NMR, 13C-NMR and ESI-MS (Suppl. S1 and S2). By comparing the chemical shifts with previous publications, we confirmed that the peak 1 is complanatosides B and the peak 2 is complanatosides A.

| 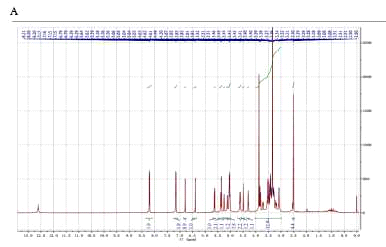 | 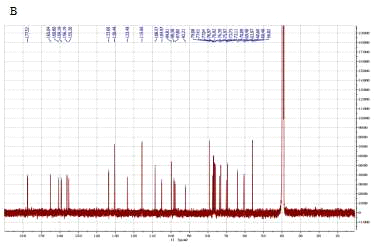 |
| --- | --- |
| 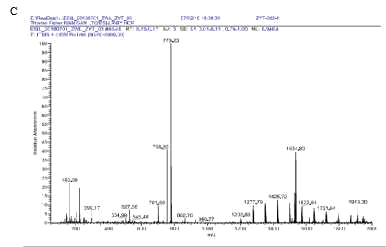 | |

**S1:** the Chromatogram of 1H-NMR, 13C-NMR and ESI-MS of compound 1. A: 1H-NMR; B:13C-NMR; C:ESI-MS.

| 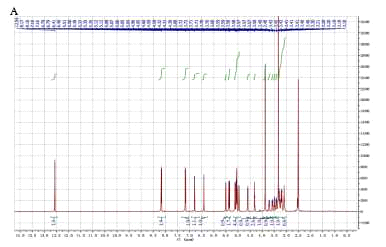 | 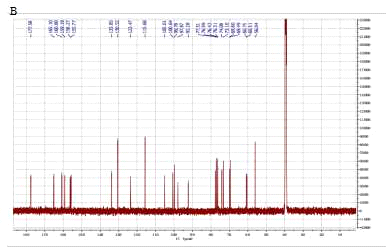 |
| --- | --- |
| 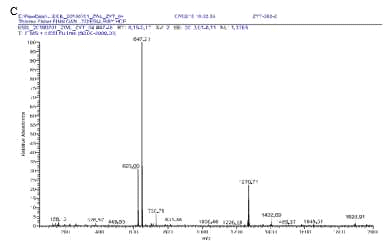 | |

**S2:** the Chromatogram of 1H-NMR, 13C-NMR and ESI-MS of compound 2. A: 1H-NMR; B:13C-NMR; C:ESI-MS.

**Materials and Methods**

2.1 Materials

NMR Brucker AM-500, 600 NMR Spectrometer were bought from Germany;Mass spectrometer Finnigan LCQ Deca XP were bought from USA. High performance liquid meter: Chromatography instrument: Dalian Elite PDA230 high pressure constant current pump, DAD230 detector. Preparative high performance liquid chromatography were produced by Suzhou Huitong Chromatography Separation and Purification Company. The Semen Astragali complanatus decoction pieces were bought from Shanghai Hua Ji Pharmaceutical Co., Ltd. (Lot: 16113004), and were grown in Shanxi Province, China.

2.2 Methods

The pieces (500g) were extracted twice with boiling water(10 L) . The decoction was combined and concentrated to about 100mL, and then loaded on DM130 macroporous resin, which was then eluted by mobile phase composed of ethanol and water from 20% to 80%. The 60–80% ethanol-eluted fraction was collected and concentrated to 200ml, then 100% ethanol was added to 1000 mL. The precipitate was removed and the upper clear liquid was concentrated to water-free, and dissolved in 20 mL of DMSO. After repeated separation on a C18 column, Compound 1 (180 mg) and Compound 2 (210 mg) were obtained.

The structures of compound 1 and compound 2 were identified by 1H-NMR, 13C-NMR and ESI-MS used Brucker AM-500, 600 NMR Spectrometer and Finnigan LCQ Deca XP respectively. Simply, for 1H-NMR and 13C-NMR, the sample were dissolved in the DMSO- d6 and loaded into the sample tube, placed the sample in the magnet and set sampling parameters. For ESI-MS, the sample were diluted with a standard solvent (50% H2O, 50% acetonitrile or methanol, 0.1% formic acid), placed the prepared sample into the injector and placed the injector in the injection pump. Then Connect the injector directly to the ion source, and set the sampling parameters. The result was shown in supply S1 and S2.
